# Trends and inequalities in the use of deworming medication during pregnancy in Sierra Leone, 2008–2019

**DOI:** 10.1186/s41182-024-00638-9

**Published:** 2024-11-04

**Authors:** Augustus Osborne, Alpha Umaru Bai-Sesay, Alieu Tommy, Camilla Bangura, Bright Opoku Ahinkorah

**Affiliations:** 1 Department of Biological Sciences, School of Basic Sciences, Njala University, PMB, Freetown, Sierra Leone; 2 Ministry of Health and Sanitation, Freetown, Sierra Leone; 3 REMS Consultancy Services, Takoradi, Sekondi-Takoradi, Ghana; 4 Faculty of Health and Medical Sciences, The University of Adelaide, Adelaide, Australia

**Keywords:** Children, Deworming, Pregnancy, Public health, Sierra Leone

## Abstract

**Background:**

Intestinal worm infections are a significant public health concern for pregnant women in low- and middle-income countries. These infections can lead to anaemia, malnutrition, and adverse pregnancy outcomes, including premature birth and low birth weight. Deworming medication during pregnancy is a safe and effective strategy to prevent these complications and improve maternal and child health. This study aims to investigate the trends and inequalities in the use of deworming medication during pregnancy among women in Sierra Leone between 2008 and 2019.

**Methods:**

The study utilised data from the Sierra Leone Demographic Health Surveys conducted in 2008, 2013, and 2019. We used the Health Equity Assessment Toolkit developed by the World Health Organisation to calculate various measures of inequality, including difference, ratio, population attributable risk, and population attributable fraction. An inequality assessment was conducted for five stratifiers: age, economic status, level of education, place of residence, and sub-national province.

**Results:**

The prevalence of deworming medication during pregnancy was 43.8% in 2008, 72.4% in 2013, and 83.5% in 2019 in Sierra Leone. There was a decrease in age-related inequality from a difference of 3.7% in 2008 to −0.8% in 2019. Economic-related inequality increased from a difference of −8.5% in 2008 to −8.2% in 2019. Both population attributable fraction and population attributable risk were zero in all survey years for economic status, indicating no improvement in the setting average without economic-related inequality. Inequality in education increased from a difference of -8.9% in 2008 to −8.4% in 2019 and decreased from a difference of −2.6% in 2008 to –5.5% in 2019 for place of residence. Provincial inequality decreased from a difference of 29.5% in 2008 to 11.8% in 2019. The population attributable risk for province reveals that the setting average could have been 10.5 percentage points lower in 2008, 8.2 percentage points lower in 2013, and 5.9 percentage points lower in 2019 without provincial inequality.

**Conclusion:**

The prevalence of deworming medication use during pregnancy substantially increased from 2008 to 2019 (43.8% to 83.5%) in Sierra Leone. This suggests a positive public health trend in maternal healthcare access and education. Inequalities related to economic status and education increased slightly while age-related, place of residence and provincial inequalities decreased. This indicates an inequitable distribution of this essential healthcare intervention across these stratifiers. The government and policymakers should continue efforts to raise awareness and promote the use of deworming medication during pregnancy.

## Introduction

Deworming during pregnancy refers to administering anthelminthic drugs to pregnant women to treat and prevent infections caused by intestinal worms, such as hookworm and *Trichuris* trichiura [1]. These infections can lead to significant health problems, including anaemia, malnutrition, and impaired foetal development [2]. A recent study found that deworming treatment during pregnancy can reduce the risk of child mortality within the first four weeks after birth by 14%, highlighting its critical importance for maternal and child health [3]. Deworming medication during pregnancy is a safe and effective strategy to prevent these complications and improve maternal and child health [4]. Ensuring that pregnant women receive deworming treatment is crucial for reducing the burden of worm infections and promoting healthier pregnancies and births [5].

Globally, the World Health Organization (WHO) emphasises the importance of deworming for pregnant women as part of its maternal health initiatives [6]. WHO estimates that a significant proportion of pregnant women in endemic areas receive deworming treatment, reflecting substantial progress, though there remains a need for continued efforts to expand coverage [7]. In sub-Saharan Africa (SSA), where intestinal worm infections are prevalent and anaemia rates among pregnant women are high, deworming during pregnancy varies widely across countries [8]. Regional disparities are evident, with East Africa reporting higher coverage (67.6%) compared to West Africa (24.3%), emphasising the uneven distribution of health interventions across the continent [8]. Deworming coverage among pregnant women in Sierra Leone increased to 83.5% in 2019, a remarkable improvement from 43.8% in 2008[9].

In Sierra Leone, the government and partner organisations have implemented several interventions and policies to address the issue of deworming during pregnancy. National campaigns and community-based programmes have increased awareness and accessibility to deworming medications. Despite these efforts, challenges such as logistical barriers, limited healthcare infrastructure, and occasional shortages of medications persist. These obstacles hinder the consistent and widespread implementation of deworming initiatives, necessitating ongoing efforts to overcome them [10].

Despite the progress made, there are still gaps in the understanding trends and inequalities in deworming during pregnancy in Sierra Leone. To the best of our knowledge, no national study has examined this issue. This study examines the trends and inequalities in deworming medication use during pregnancy in Sierra Leone from 2008 to 2019. This research will contribute significantly to developing targeted interventions and policies by identifying disparities and areas needing improvement. The findings will be valuable to the government, policymakers, healthcare providers, and researchers, enhancing maternal health programmes and the well-being of pregnant women in Sierra Leone.

## Methods

### Study design and source

We utilised data from the 2008, 2013, and 2019 Sierra Leone Demographic Health Survey (SLDHS). The SLDHS is a comprehensive survey conducted over the entire country to identify patterns and fluctuations in demographic indicators, health indicators, and social issues among individuals of all genders and age groups. The study employed a cross-sectional design, selecting individuals through a stratified multi-stage cluster sampling method. The SLDHS report comprehensively explains the sampling process [9]. This study included pregnant women aged 15 and 49 participating in the SLDHS. The 2008, 2013, and 2019 SLDHS data were accessible for immediate utilisation through the WHO HEAT online platform [10]. This study was carefully designed, considering the standards outlined in the Strengthening the Reporting of Observational Studies in Epidemiology (STROBE) statement [11].

### Variables

The utilisation of deworming medication use during pregnancy was the dependent variable. According to the WHO, it is advised that a pregnant woman should take a single dosage of either mebendazole (500 mg) or albendazole (400 mg) after the first three months of pregnancy [8, 13, 14]. During the DHS survey, women who were of reproductive age and had given birth before the survey were asked if they had used deworming medicine. The women were allowed to respond with either a yes or a no [15, 16]. The dependent variable was dichotomously coded as “yes” if the women took deworming medicine and “no” if they did not. The evaluation of disparity in deworming medication use was conducted using five stratifiers: age (15–19, 20–24, 25–29, 30–34, 35–39, 40–44, and 45–49), economic status determined by wealth quintile (quintile 1, 2, 3, 4, 5), educational attainment (no education, primary education, secondary and higher education), residential location (rural, urban), and sub-national province (Eastern, Northern, Northwestern, Southern, Western).

### Data analysis

The analysis was conducted using the online version of the WHO HEAT programme [11]. Estimates and confidence intervals (CIs) were calculated to evaluate the deworming medication use during pregnancy among women, considering the stated stratifiers. Four metrics were employed to calculate inequality: difference (D), population attributable risk (PAR), population attributable fraction (PAF), and ratio (R). Two basic measurements, D and R, are weight-independent, but two more complex measures, PAR and PAF, are weight-dependent. R and PAF are relative metrics used to compare and evaluate different factors about each other. Nevertheless, D and PAR are absolute metrics. The consideration of summary measures is based on the acknowledgement by the WHO that both absolute and relative summary measurements are crucial for obtaining policy-oriented conclusions. Unlike basic measurements, complex measures consider the magnitude of categories within a particular population subset. The literature has thoroughly explained the WHO’s summary measurements and calculations [17, 18].

## Results

Table 1 shows a positive trend in the use of deworming medication during pregnancy across various inequality dimensions in Sierra Leone between 2008 and 2019. The national prevalence of deworming medication during pregnancy was 43.8% in 2008, 72.4% in 2013, and 83.5% in 2019. There was an apparent increase in deworming medication use across all age groups. However, the increase seems slightly higher for younger women (15–19 years) than older ones (40–49 years). Similar to age, all economic quintiles showed increased deworming medication use. Interestingly, the poorest quintile (Quintile 1) started with the lowest prevalence and ended with a value close to the richest (Quintile 5). All education levels showed an increase in deworming medication use. Notably, women with higher education (secondary and higher) started with a higher prevalence in 2008 and maintained that lead throughout the period. Both rural and urban areas showed an increase (rural: 43.0% in 2008, 71.0% in 2013, and 81.4% in 2019 versus urban: 45.7% in 2008, 75.9% in 2013, and 86.9% in 2019), with urban areas starting and ending with a slightly higher prevalence. All provinces showed an increase in deworming medication use overtime.

**Table 1:** Trends in the prevalence of women who took deworming medication during pregnancy of last birth by different inequality dimensions in Sierra Leone, 2008–2019

	2008(43.8%)		2013 (72.4%)		2019 (83.5%)		
Dimension	Est	N	Est	N		Est	N
Age							
15–19 years	43.7	330	68.6	859		82.5	598
20–24 years	47.2	804	71.8	1773		80.5	1611
25–29 years	42.4	1213	74.5	2142		85.6	1910
30–34 years	41.4	704	73.5	1644		83.6	1309
35–39 years	46.0	673	71.1	1354		83.5	1234
40–44 years	42.5	251	71.6	554		85.5	454
45–49 years	40.0	127	71.8	322		83.3	211
Economic status							
Quintile 1 (poorest)	40.2	885	67.8	1901		81.3	1587
Quintile 2	42.4	849	70.9	1809		81.3	1551
Quintile 3	43.2	893	72.5	1797		80.6	1487
Quintile 4	45.7	793	74.0	1694		86	1441
Quintile 5 (richest)	48.7	683	78.1	1447		89.5	1259
Education							
No education	41.5	3051	70	5768		82.1	3857
Primary education	49.2	515	76.1	1203		82.0	1033
Secondary education	52.0	482	77.5	1559		85.8	2214
Higher education	50.4	55	82.9	117		90.5	221
Residence							
Rural	43.0	2920	71.0	6260		81.4	4531
Urban	45.7	1183	75.9	2387		86.9	2795
Province							
Eastern	33.2	809	64.0	2054		77.5	1542
Northern	39	1869	72.9	4982		83.4	1433
Northwestern	NA	NA	NA	NA		79.4	1380
Southern	62.7	783	81.4	1611		87.6	1492
Western	48.29	642	NA	NA		89.4	1479

*Est* estimate, *N *sample size, *NA* not available as between 2008 and 2013, Sierra Leone had four regions

**Fig. 1:**
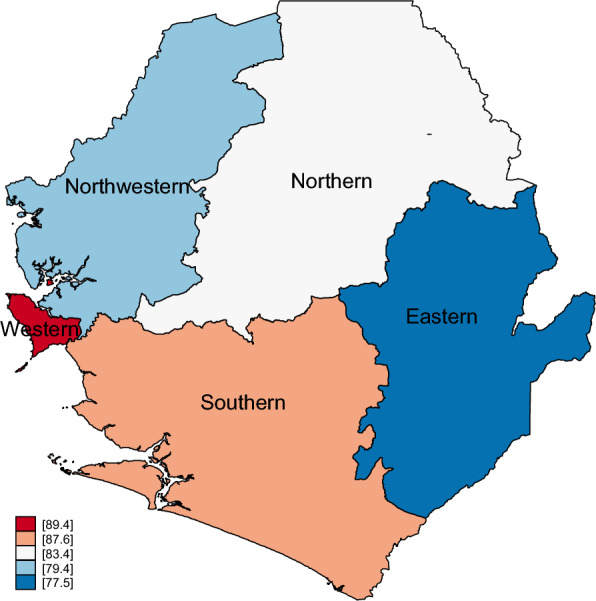
Provincial prevalence (%) of women who took deworming medication during pregnancy of last birth in Sierra Leone in 2019

Figure 1 shows the provincial prevalence of women who took deworming medication during pregnancy in Sierra Leone in 2019. The Western province had the highest prevalence of 89.4% of women who took deworming medicines during pregnancy in Sierra Leone, whilst the Eastern province had the lowest prevalence of 77.5% of women who took deworming medication during pregnancy in Sierra Leone.

Table 2 shows inequality indices for women who took deworming medication during pregnancy in Sierra Leone from 2008 to 2019. There was a decrease in age-related inequality from 3.7% in 2008 to −0.8% in 2019 (D=3.7 in 2008 and −0.8 in 2019). The PAR reveals that the setting average could have been 3.7 percentage points lower in 2008, 0.6 percentage points lower in 2013, and 0.2 percentage points lower in 2019 without age inequality. Economic-related inequality increased from −8.5% in 2008 to −8.2% in 2019. Both PAF and PAR were zero in all survey years, indicating no further improvement can be achieved in the setting average without economic-based inequality. Inequality in education increased from −8.9% in 2008 to −8.4% in 2019. PAF and PAR were zero in 2008, 2013 and 2019, indicating that no further improvement can be achieved in the setting average in the absence of education-related inequality. Inequality decreased from −2.6% in 2008 to −5.5% in 2019 for place of residence. PAF and PAR were zero in 2008, 2013 and 2019, indicating that no further improvement can be achieved in the setting average in the absence of residence-related inequality. Provincial inequality decreased slightly from 29.5% in 2008 to 11.8% in 2019. The PAR reveal that the setting average could have been 10.5 percentage points lower in 2008, 8.2 percentage points lower in 2013, and 5.9 percentage points lower in 2019 without provincial inequality.

**Table 2:** Inequality indices of estimates of factors associated with women who took deworming medication during pregnancy of last birth in Sierra Leone, 2008–2019

	2008			2013			2019		
Dimension	Est	LB	UB	Est	LB	UB	Est	LB	UB
Age									
D	3.7	NA	NA	−3.1	NA	NA	−0.8	NA	NA
PAF	−8.5	−8.7	−8.3	−0.8	−0.8	−0.8	−0.2	−0.3	−0.2
PAR	−3.7	−12.1	4.6	−0.6	−2.4	1.2	−0.2	−5.1	4.7
R	1.0	NA	NA	0.9	NA	NA	0.9	NA	NA
Economic status									
D	−8.5	NA	NA	−10.3	NA	NA	−8.2	NA	NA
PAF	0	−0.0	0.0	0	−0.0	0.0	0	−0.0	0.0
PAR	0	−3.4	3.4	0	−1.9	1.9	0	−1.6	1.6
R	0.8	NA	NA	0.8	NA	NA	0.9	NA	NA
Education									
D	−8.9	NA	NA	−12.9	NA	NA	-8.4	NA	NA
PAF	0	−0.2	0.2	0	−0.0	0.0	0	−0.0	0.0
PAR	0	−13.1	13.1	0	−6.8	6.8	0	−3.8	3.8
R	0.8	NA	NA	0.8	NA	NA	0.9	NA	NA
Residence									
D	−2.6	NA	NA	−4.8	NA	NA	−5.5	NA	NA
PAF	0	−0.0	0.0	0	−0.0	0.0	0	−0.0	0.0
PAR	0	−2.3	2.3	0	−1.4	1.4	0	−1.0	1.0
R	0.9	NA	NA	0.9	NA	NA	0.9	NA	NA
Province									
D	29.5	NA	NA	17.3	NA	NA	11.8	NA	NA
PAF	−24.0	−24.1	−24.0	−11.4	−11.4	−11.4	−7.0	−7.1	−7.0
PAR	−10.5	−13.5	−7.6	−8.2	−10.0	−6.5	−5.9	−7.7	−4.1
R	1.8	NA	NA	1.2	NA	NA	1.1	NA	NA

*Est* estimate, *UB* upper bound, and *LB* lower bound, *D* difference, *NA* not available, *PAF* population attributable fraction, *PAR* population attributable risk, *R* ratio

## Discussion

This study used nationally representative data to examine the trends and inequalities in deworming medicine use during pregnancy in Sierra Leone from 2008 to 2019. We found that deworming medication use during pregnancy increased from 43.8% in 2008 to 83.5% in 2019, with age, place of residence, provincial inequalities decreasing, and economic and education inequalities showing slight increases.

We found that Sierra Leone continues to have a high deworming drug uptake as the proportion of pregnant women using deworming medicine almost doubled from 2008 to 2019. This increase may have resulted from possible public health campaigns and programmes that aimed to improve maternal health in Sierra Leone. The rise in prevalence also highlights the continued work required to close gaps and achieve universal coverage [10]. According to the WHO approximately 50% of pregnant women in endemic areas receive deworming treatment, reflecting progress, but highlighting ongoing challenges in achieving universal coverage [6]. The result of our finding is higher than the previous research conducted in Benin, which showed that 35% of pregnant women there used deworming medicine, indicating a limited medication uptake [5]. Age-related inequality decreased from a difference of 3.7 per cent in 2008 to -0.8 per cent in 2019, which is a sign that efforts to achieve age equity in the usage of deworming medications are making headway. Age inequality had a decreasing effect on the overall setting average. This implies that reasonably successful attempts to target both older and younger pregnant women have resulted in a more consistent distribution of deworming medication use across age groups. Sustaining and advancing this trend will require ongoing observation and customised actions.

Women across all educational levels showed increased use of deworming medication, with those having higher education (secondary and higher) maintaining a consistently higher prevalence from 2008 to 2019. According to previous research, the use of deworming medication in SSA has been associated with increased educational attainment [5, 19]. One possible explanation might be that access to healthcare services and the intention to seek health are significantly influenced by one's level of education [20]. Educated women are more likely to navigate healthcare systems and comprehend the advantages of deworming successfully. The findings from our study highlight the need for strategies that not only increase educational opportunities for women, but also enhance health literacy and awareness about deworming benefits across all educational levels. Community-based education programmes, integration of health topics into formal education, and targeted awareness campaigns could help bridge this gap. Economic inequality increased somewhat from a difference of −8.5 per cent in 2008 to −8.2 per cent in 2019 indicating that women from lower economic status had lower coverage. This study is in line with the previous study in Tanzania, which found that wealthier women are more likely to use deworming drugs than the poor [22]. The finding indicates that policy efforts targeting the poorest quintiles are needed and more steps are required to address economic inequities actively to make more significant gains. These steps could include expanding financial assistance, enhancing low-income populations' access to services, and implementing extensive community health programmes.

The provincial inequality in deworming medication use during pregnancy decreased in Sierra Leone between 2008 and 2019. Deworming medication is often provided during antenatal care (ANC) visits. Increased access to ANC services in provinces could have led to a more even medication distribution. Public health campaigns aimed explicitly at provinces with lower deworming rates can raise awareness about the benefits of the medication and encouraged pregnant women to seek it out. Investments in healthcare infrastructure, such as new clinics or mobile health units in remote areas, can make deworming medication more readily available nationwide. Local community education initiatives involving traditional birth attendants, community health workers, and peer educators can help improve knowledge about deworming medication and addressed cultural barriers to its use.

### Policy and practice implications

The increase in the national prevalence of deworming medication use during pregnancy over time suggests existing policies and practices might be working. Continued funding and support for these programmes are likely warranted. Policies should continue to focus on integrating deworming medication information into existing educational programmes for women of all ages. Collaborating with the Ministry of Education to develop curriculum materials could be beneficial. Provincial inequalities require targeted interventions and policies could include increased allocation of resources to remote areas, such as deploying mobile clinics or establishing community health worker programmes and collaborating with local leaders to address cultural barriers or logistical challenges in accessing healthcare in specific provinces. The study highlights the importance of ongoing monitoring and evaluation to assess existing policies’ effectiveness and identify areas for improvement. Regular data collection on deworming medication use, disaggregated by relevant factors like education and province, is crucial for informed policy decisions.

### Strengths and limitations

The SLDHS provides a comprehensive and representative dataset on women's health in Sierra Leone. This allows for generalisable conclusions about trends and inequalities in deworming medication use. Data availability from 2008 to 2019 enabled analysis of trends in deworming medication use over time. The SLDHS collects data on various factors, such as age, economic status, education, and residence. This allowed for investigating inequalities in deworming medication use across these categories. HEAT provides tools for analysing and visualising health inequality data. This helped present the study's findings clearly and compellingly. Despite these strengths, there are some limitations that need to be acknowledged. First, the data on deworming medication use are based on self-reported information from women, which may be subject to recall bias. Again, the SLDHS does not collect clinical data on women's health or the effectiveness of deworming medication. These could have helped to provide further explanations to some of our findings.

## Conclusion

The prevalence of deworming medication use during pregnancy substantially increased from 2008 to 2019 (43.8% to 83.5%). This suggests a positive public health trend in maternal healthcare access and education. Despite the increase, inequalities in access to deworming medication persist, mainly based on economic status and education. The government and policymakers should continue efforts to raise awareness and promote the use of deworming medication during pregnancy; develop targeted interventions to address these disparities, such as educational campaigns tailored to women with lower education levels; culturally appropriate messaging and education materials, and monitoring and evaluation of interventions' effectiveness to ensure they achieve the desired outcomes. By implementing these recommendations and conducting further research, Sierra Leone can continue to improve access to deworming medication during pregnancy and ensure equitable maternal healthcare for all women.

## Data Availability

The dataset used can be accessed at https://whoequity.shinyapps.io/heat/

## Abbreviations

ANC: Antenatal care
D: Difference
HEAT: Health Equity Assessment Toolkit
DHS: Demographic health survey
PAF: Population attributable fraction
PAR: Population attributable risk
R: Ratio
SSA: Sub-Saharan Africa
WHO: World Health Organization
